# The genome sequence of the Water scavenger beetle, *Sphaeridium bipustulatum* Fabricius, 1781 (Coleoptera: Hydrophilidae)

**DOI:** 10.12688/wellcomeopenres.27731.1

**Published:** 2026-09-07

**Authors:** František Sládeček, Liam M. Crowley, University of Oxford and Wytham Woods Genome Acquisition Lab, Darwin Tree of Life Barcoding Collective, Wellcome Sanger Institute Tree of Life Management, Samples and Laboratory team, Wellcome Sanger Institute Scientific Operations: Sequencing Operations, Wellcome Sanger Institute Tree of Life Core Informatics team, Tree of Life Core Informatics collective, Darwin Tree of Life Consortium

**Affiliations:** 1 University of South Bohemia, České Budějovice, South Bohemia, Czech Republic; 2 University of Oxford, Oxford, Oxfordshire, England, UK

**Keywords:** Sphaeridium bipustulatum, Water scavenger beetle, genome sequence, chromosomal, Coleoptera

## Abstract

We present a genome assembly from an individual male *Sphaeridium bipustulatum* (Water scavenger beetle; Arthropoda; Insecta; Coleoptera; Hydrophilidae). The primary assembly has a total length of 261.74 megabases. Most of the primary assembly (99.19%) is scaffolded into 13 chromosomal pseudomolecules, including the X and Y sex chromosomes. The mitochondrial genome has also been assembled, with a length of 16.79 kilobases. This assembly was generated as part of the Darwin Tree of Life project, which produces genomes for eukaryotic species found in Britain and Ireland.

## Species taxonomy

Eukaryota; Opisthokonta; Metazoa; Eumetazoa; Bilateria; Protostomia; Ecdysozoa; Panarthropoda; Arthropoda; Mandibulata; Pancrustacea; Altocrustacea; Allotriocarida; Hexapoda; Insecta; Dicondylia; Pterygota; Neoptera; Eumetabola; Endopterygota; Aparaglossata; Neuropteroidea; Coleoptera; Polyphaga; Staphyliniformia; Hydrophiloidea; Hydrophilidae; Sphaeridiinae; *Sphaeridium*; *Sphaeridium bipustulatum* Fabricius, 1781 (NCBI:txid290649)

## Background

*Sphaeridium bipustulatum* Fabricius, 1781 is a rounded, glossy beetle of the family Hydrophilidae, measuring 4.5–6.3 mm in length. The body is predominantly black, with reddish markings towards the rear of the elytra and paler margins to the pronotum. The elytra are shiny, with distinct rows of punctures and a smooth surface between them ( Berge Henegouwen & Foster, 2019). Although the Hydrophilidae are mainly aquatic, *Sphaeridium* belongs to the largely terrestrial subfamily Sphaeridiinae, many members of which live in decaying organic material and mammalian dung ( Greń & Górz, 2020).

*Sphaeridium bipustulatum* is widely distributed in the Palaearctic and has also been introduced to North America. It is associated with the dung of herbivorous mammals, particularly even-toed ungulates ( Greń & Górz, 2020). Fresh cow pats are rapidly colonised by *Sphaeridium*, and studies of other species in the genus show that their larvae are carnivorous, feeding mainly on fly maggots. Females lay their eggs in fresh dung and the larvae develop rapidly; when prey is scarce, larvae may also prey on other *Sphaeridium* larvae ( Sowig, 1997).

Identification of *S. bipustulatum* has historically been complicated by its similarity to *S. marginatum*, which was formerly treated as a form of *S. bipustulatum* before being reinstated as a separate species in 1989 ( Berge Henegouwen & Foster, 2019).

We present a chromosome-level genome sequence for *Sphaeridium bipustulatum*. produced using the Tree of Life pipeline. It was generated as part of the Darwin Tree of Life Project, which aims to generate high-quality reference genomes for all named eukaryotic species in Britain and Ireland to support research, conservation, and the sustainable use of biodiversity ( Darwin Tree of Life Project Consortium, 2022). The sequence is based on a specimen collected from Blackdown Farm, Gloucestershire, UK (Figure 1).

**Figure 1.**
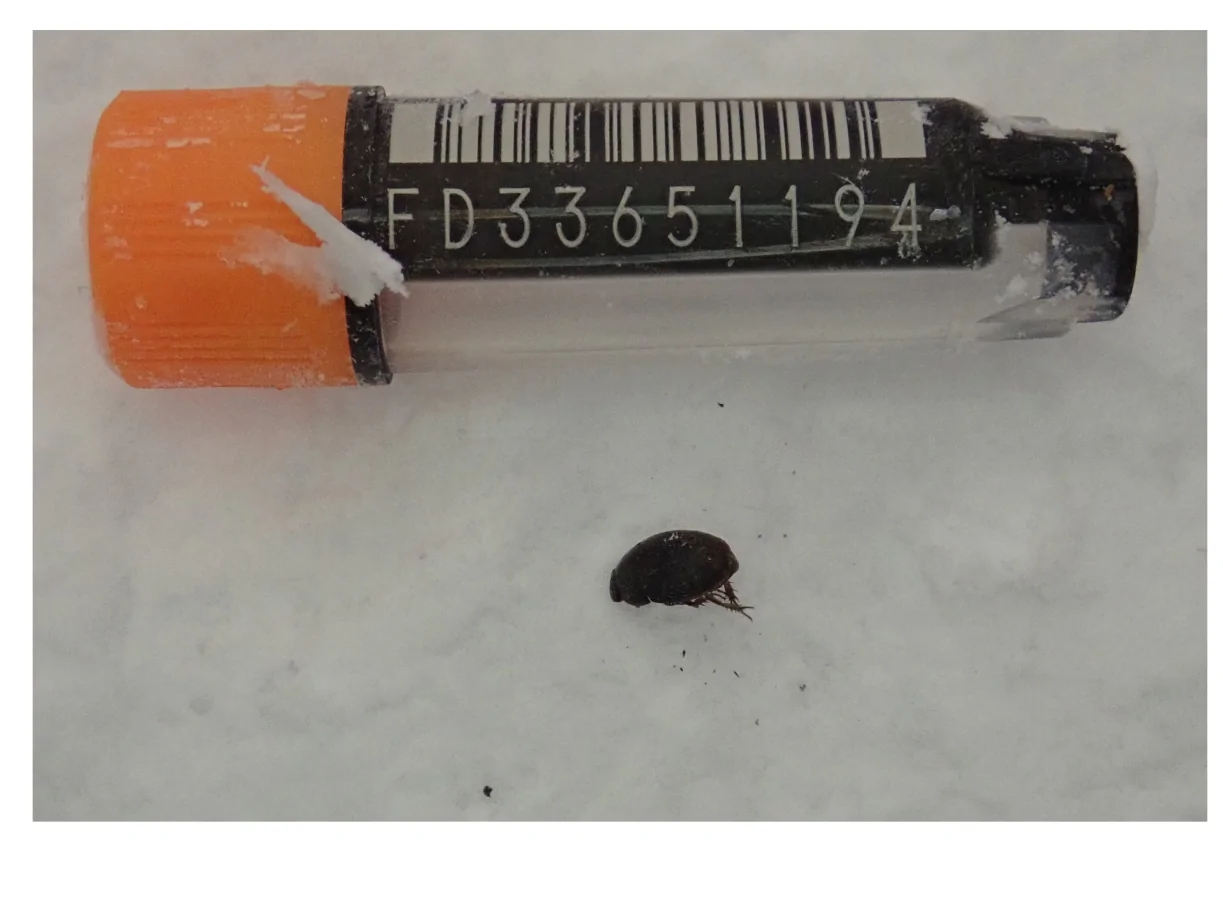
The *S. bipustulatum* specimen (Ox002969, icSphBipu4) used for genome sequencing.

## Methods

### Sample acquisition and DNA barcoding

Three adult *Sphaeridium bipustulatum* specimens were used: the male genome-sequencing specimen (specimen ID Ox002969, ToLID icSphBipu4; Figure 1), the Hi-C specimen (specimen ID Ox000257, ToLID icSphBipu2), and the RNA specimen (specimen ID Ox002970, ToLID icSphBipu5). The genome-sequencing specimen was collected from Blackdown Farm, Gloucestershire, UK (latitude 52.042, longitude −1.696) on 2022-08-25 by František Sládeček and Liam Crowley and identified by František Sládeček. The Hi-C specimen was collected from Wytham Farm, UK (latitude 51.784, longitude −1.31) on 2019-09-03 by František Sládeček, who also identified it. The RNA specimen was collected from Blackdown Farm, Gloucestershire, UK (latitude 52.042, longitude −1.696) on 2022-08-25 by František Sládeček and Liam Crowley and identified by František Sládeček.

The initial identification was verified by an additional DNA barcoding process according to the framework developed by Twyford *et al.*. (2024). A small sample was dissected from the specimen and stored in ethanol, while the remaining parts were shipped on dry ice to the Wellcome Sanger Institute (WSI) (see the protocol). The tissue was lysed, the COI marker region was amplified by PCR, and amplicons were sequenced and compared to the BOLD database, confirming the species identification ( Crowley *et al.* , 2023). Following whole genome sequence generation, the relevant DNA barcode region was also used alongside the initial barcoding data for sample tracking at the WSI ( Twyford *et al.* , 2024). The standard operating procedures for Darwin Tree of Life barcoding are available on protocols.io.

### Nucleic acid extraction

High molecular weight (HMW) DNA extraction protocols developed by the Tree of Life Core Laboratory at the Wellcome Sanger Institute (WSI) are available on protocols.io ( Howard *et al.*, 2025). Tissue from the icSphBipu4 sample was weighed and triaged to select the appropriate extraction workflow. HMW DNA was prepared for one PacBio HiFi library: DTOL15273247, for which 5.0 mg of whole organism tissue was used. Tissue from the whole organism was homogenised by powermashing using a PowerMasher II tissue disruptor. HMW DNA was extracted using the manual MagAttract v2 workflow. DNA was sheared into an average fragment size of 12–20 kb following the Megaruptor®3 for LI PacBio protocol. Sheared DNA was purified by Automated SPRI.

The concentration of the sheared and purified DNA was assessed with a NanoDrop spectrophotometer and a Qubit Fluorometer using the Qubit dsDNA High Sensitivity Assay kit. Fragment size distribution was evaluated by running the sample on the Femto Pulse system. For this sample, final post-shearing DNA measurements included a Qubit concentration of 3.16 ng/μL, and an average fragment size of 8.3 kb.

RNA was extracted from whole organism tissue of the icSphBipu5 specimen in the Tree of Life Laboratory at the WSI using the RNA Extraction: Automated MagMax™ *mir*Vana protocol. The RNA concentration was assessed with a NanoDrop spectrophotometer and a Qubit Fluorometer using the Qubit RNA Broad-Range Assay kit. RNA integrity was assessed using the Agilent RNA 6000 Pico Kit and Eukaryotic Total RNA assay.

### PacBio HiFi library preparation and sequencing

Library preparation and sequencing took place in the WSI Scientific Operations core. Library DTOL15273247 was prepared with the SMRTbell Prep Kit 3.0 (Pacific Biosciences) according to the manufacturer’s instructions. The kit includes reagents for end repair/A-tailing, adapter ligation, post-ligation SMRTbell bead clean-up, and nuclease treatment. Size selection and clean-up were performed using diluted AMPure PB beads (Pacific Biosciences). DNA concentration was quantified with a Qubit Fluorometer v4.0 (Thermo Fisher Scientific) and the Qubit 1X dsDNA HS assay kit. Final library fragment size was assessed with the Agilent Femto Pulse Automated Pulsed Field CE Instrument (Agilent Technologies) using the gDNA 55 kb BAC analysis kit.

The library was sequenced on a Revio instrument (Pacific Biosciences, California, USA). The prepared libraries selected for multiplexing were pooled based on genome size, library molarity and the intended plex level. Primers were annealed and polymerases were bound to generate circularised complexes following the manufacturer’s instructions. Complexes were purified using SMRTbell beads, diluted to the Revio loading concentration, and spiked with a Revio sequencing internal control. The pooled libraries were sequenced on a Revio 25M Plex SMRT cell in a 2-plex run. SMRT Link software (Pacific Biosciences) was used to configure and monitor the run and to perform primary and secondary data analysis.

### Hi-C

***Sample preparation and crosslinking***

Hi-C data were generated from the icSphBipu2 whole organism sample using the Arima-HiC v2 kit (Arima Genomics). Following the manufacturer’s instructions, tissue was fixed and DNA crosslinked using TC buffer to a final formaldehyde concentration of 2%. The tissue was homogenised using the Diagnocine Power Masher-II. Crosslinked DNA was digested with a restriction enzyme master mix, biotinylated, and ligated. Clean-up was performed with SPRISelect beads before library preparation. DNA concentration was measured with the Qubit Fluorometer (Thermo Fisher Scientific) and Qubit HS Assay Kit. The biotinylation percentage was estimated using the Arima-HiC v2 QC beads.

***Hi-C library preparation and sequencing***

Biotinylated DNA constructs were fragmented using a Covaris E220 sonicator and size-selected to 400–600 bp using SPRISelect beads. DNA was enriched with Arima-HiC v2 kit Enrichment beads. End repair, A-tailing, and adapter ligation were carried out with the NEBNext Ultra II DNA Library Prep Kit (New England Biolabs), following a modified protocol where library preparation occurs while DNA remains bound to the Enrichment beads. Libraries were amplified with KAPA HiFi HotStart mix and a custom Unique Dual Index (UDI) barcode set (Integrated DNA Technologies). Depending on sample concentration and biotinylation percentage determined at the crosslinking stage, libraries were amplified with 10–16 PCR cycles. Post-PCR clean-up was performed with SPRISelect beads. Libraries were quantified using the AccuClear Ultra High Sensitivity dsDNA Standards Assay Kit (Biotium) and a FLUOstar Omega plate reader (BMG Labtech).

Prior to sequencing, libraries were normalised to 10 ng/μL. Normalised libraries were quantified again to create equimolar and/or weighted 2.8 nM pools. Pool concentrations were checked using the Agilent 4200 TapeStation (Agilent) with High Sensitivity D500 reagents before sequencing. Libraries were sequenced on the Illumina NovaSeq 6000, generating paired-end 150-bp reads.

### RNA library preparation and sequencing

Libraries were prepared with the NEBNext® Ultra II Directional RNA Library Prep Kit for Illumina (New England Biolabs), following the manufacturer’s instructions. Poly(A) mRNA was isolated from 100 ng of total RNA using oligo(dT) beads, converted to cDNA, and uniquely indexed; 14 PCR cycles were performed. Libraries were prepared with an intended average fragment size of 100–300 bp. Libraries were quantified, normalised, and pooled equimolarly to a final concentration of 2.8 nM before dilution to 150 pM for loading. Sequencing was carried out on the Illumina NovaSeq X, generating paired-end reads.

### Genome assembly

Prior to assembly of the PacBio HiFi reads, a database of *k*-mer counts ( *k* = 31) was generated from the filtered reads using FastK. The *k*-mer frequency distribution was analysed with GenomeScope (version 2.1.0) ( Ranallo-Benavidez *et al.*, 2020), providing fitted point estimates of genome size, heterozygosity and repeat content.

The HiFi reads were assembled using Hifiasm ( Cheng *et al.*, 2021) with the --primary option. The Hi-C reads ( Rao *et al.* , 2014) were mapped to contigs using bwa-mem2 ( Vasimuddin *et al.*, 2019), and the contigs were scaffolded in YaHS ( Zhou *et al.*, 2023) with the --break option for handling potential misassemblies. The scaffolded assemblies were evaluated using Gfastats ( Formenti *et al.* , 2022), BUSCO ( Manni *et al.*, 2021) and MerquryFK ( Rhie *et al.*, 2020).

The mitochondrial genome was assembled using MitoHiFi ( Uliano-Silva *et al.* , 2023). The MitoHiFi reference was the mitochondrial genome of *Sphaeridium bipustulatum* (NC_028612.1).

### Assembly curation

The assembly was decontaminated using the Assembly Screen for Cobionts and Contaminants ( ASCC) pipeline. The flat files and maps for curation were generated with TreeVal. Manual curation was conducted primarily in PretextView and HiGlass ( Kerpedjiev *et al.* , 2018). Scaffolds were visually inspected and corrected as described by Howe *et al*. (2021). The primary and alternate assemblies were combined for curation. Manual corrections included 30 breaks and 52 joins, which reduced the scaffold count by 3.8%. The curation process is described at sanger-tol/curation-resources. A Hi-C contact map of the primary assembly was generated with PretextSnapshot.

### Assembly quality assessment

The MerquryFK tool ( Rhie *et al.*, 2020) was run in a Singularity container ( Kurtzer *et al.*, 2017) to evaluate *k*-mer completeness and assembly quality for the primary and alternate haplotypes using the *k*-mer database ( *k* = 31) computed prior to genome assembly. The analysis outputs included assembly QV scores and completeness statistics.

The primary assembly was assessed using BlobToolKit ( Challis *et al.*, 2020). PacBio reads were mapped to the primary assembly to derive sequence coverage, and BUSCO ( Manni *et al.*, 2021) was used to assess gene-set completeness. DIAMOND BLASTp searches of extracted BUSCO proteins and DIAMOND BLASTx searches of assembly sequences against UniProt Reference Proteomes ( Buchfink *et al.*, 2021), followed by BLASTn searches of sequences without DIAMOND BLASTx hits against the NCBI nt database ( Altschul *et al.*, 1990), supported taxonomic assignment. Coverage, sequence composition, taxonomic assignment and BUSCO results were integrated into a BlobDir for interactive inspection and visualisation. The snail and blob plots were rendered locally from this hosted BlobDir using BlobTk v0.8.3. The scaffold-length radial scale function for the snail plot was set to linear ( Challis & Blaxter, 2026). The blob plot used circular markers with area proportional to scaffold length ( Challis *et al.*, 2020).

Ancestral linkage groups were painted along the chromosome-level scaffolds. Complete BUSCO genes (single-copy and duplicated) from the coleoptera_odb12 lineage dataset were assigned to the 8 Coleoptera ancestral linkage groups (C1–C7 and CX) described by Maulana *et al.*. (2026) and plotted along chromosomes drawn to scale.

## Genome sequence report

### Sequence data

PacBio sequencing of the *S. bipustulatum* specimen generated 13.93 Gb (gigabases) from 2.13 million reads. We assembled the genome from these reads. GenomeScope analysis using a *p* = 2 model gave a *k*-mer-based haploid genome size estimate of 260.98 Mb, with heterozygosity of 1.61% and repeat content of 17.23%; the full-model fit was 96.03% (Figure 2). The GenomeScope estimates guided expectations for the assembly. The sequencing data provided approximately 51× coverage of the primary assembly. Hi-C sequencing produced 101.97 Gb from 337.66 million reads, which were used to scaffold the primary assembly. RNA sequencing data were also generated and are available in public sequence repositories. Table 1 summarises the specimen and sequencing details.

**Figure 2.**
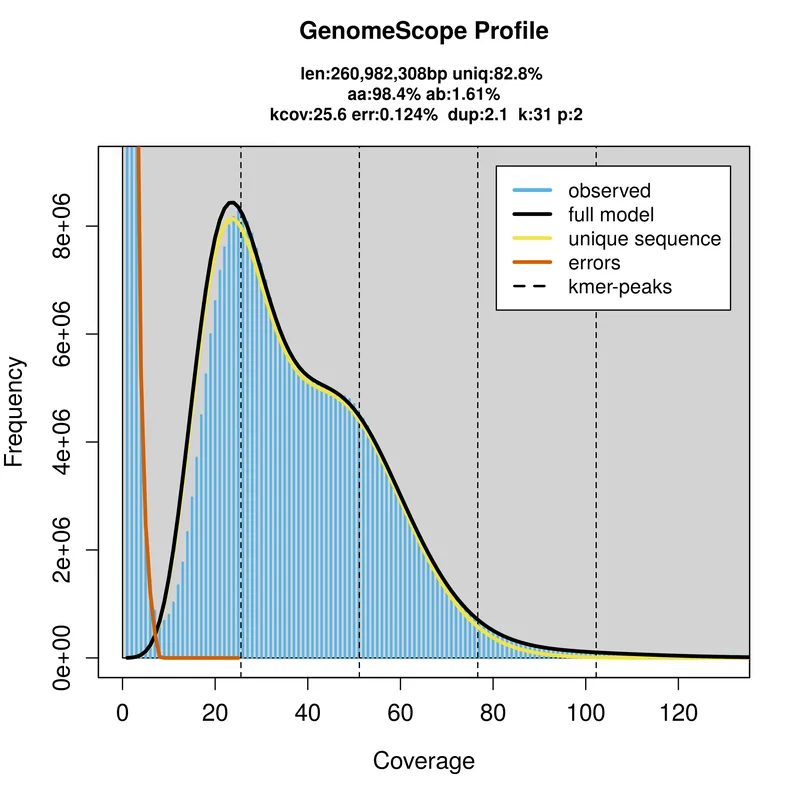
GenomeScope model fitted to the *k*-mer frequency distribution. The plot shows the observed and modelled *k*-mer spectra used to estimate genome size, heterozygosity and repeat content from unassembled sequencing reads.

**Table 1.** Specimen and sequencing data for *S. bipustulatum* (BioProject PRJEB92312)

**Platform**	**PacBio HiFi**	**Hi-C**	**RNA-seq**
**ToLID**	icSphBipu4	icSphBipu2	icSphBipu5
**Specimen ID**	Ox002969	Ox000257	Ox002970
**BioSample (source individual)**	SAMEA112774817	SAMEA7520025	SAMEA112774818
**BioSample (tissue)**	SAMEA112774911	SAMEA7520103	SAMEA112774912
**Tissue**	whole organism	whole organism	whole organism
**Instrument**	Revio (1 run)	Illumina NovaSeq 6000	Illumina NovaSeq X
**Library ID & run accession(s)**	DTOL15273247: ERR15282593	ERR15286579	ERR17468150
**Read count total**	2.13 million reads	337.66 million read pairs	48.13 million read pairs
**Base count total**	13.93 Gb	101.97 Gb	14.53 Gb

### Assembly statistics

The primary assembly was generated and an alternate haplotype assembly was also deposited at scaffold level in INSDC databases. The primary assembly is proposed as the reference assembly for this species. It has a total length of 261.74 Mb in 126 scaffolds (excluding organelle sequences), with 181 gaps, and a scaffold N50 of 22.9 Mb (Table 2). The primary assembly has a scaffold auN of 23.9 Mb.

**Table 2.** Genome assembly statistics for *S. bipustulatum*

**Genome assembly**	**Primary assembly**	**Alternate haplotype assembly**
**Assembly name**	icSphBipu4.1	icSphBipu4.1 alternate haplotype
**Assembly accession**	GCA_977013595.1	GCA_977013195.1
**Assembly level**	chromosome	scaffold
**Span (Mb)**	261.74	280.56
**Number of chromosomes**	13	-
**Number of contigs**	306	7,503
**Contig N50 (Mb)**	4.12	0.13
**Number of scaffolds (excluding organelle sequences)**	126	5,648
**Scaffold N50 (Mb)**	22.9	17.78
**Scaffold auN (Mb)**	23.9	-
**Longest scaffold length (Mb)**	33.58	28.3
**Sex chromosomes**	X and Y	-
**Organelles**	Mitochondrial genome: 16.79 kb	-

Most of the primary assembly sequence (99.19%) was assigned to 13 chromosome-level scaffolds, representing 11 autosomes and the X and Y sex chromosomes. These chromosome-level scaffolds, confirmed by Hi-C data, are named according to size (Figure 3; Table 3). During genome curation, sex chromosomes were assigned based on the copy number on the diploid HiC map. The PacBio and Hi-C data were from different individuals, so some variations and lack of Hi-C signal could be observed. We also observed a haplotypic inversion on Chromosome 11 from 1.45 to 5.86 Mbp.

The mitochondrial genome was also assembled (length 16.79 kb, OZ350008.1). This sequence is included as a contig in the multifasta file of the primary genome submission and as a standalone record.

**Figure 3.**
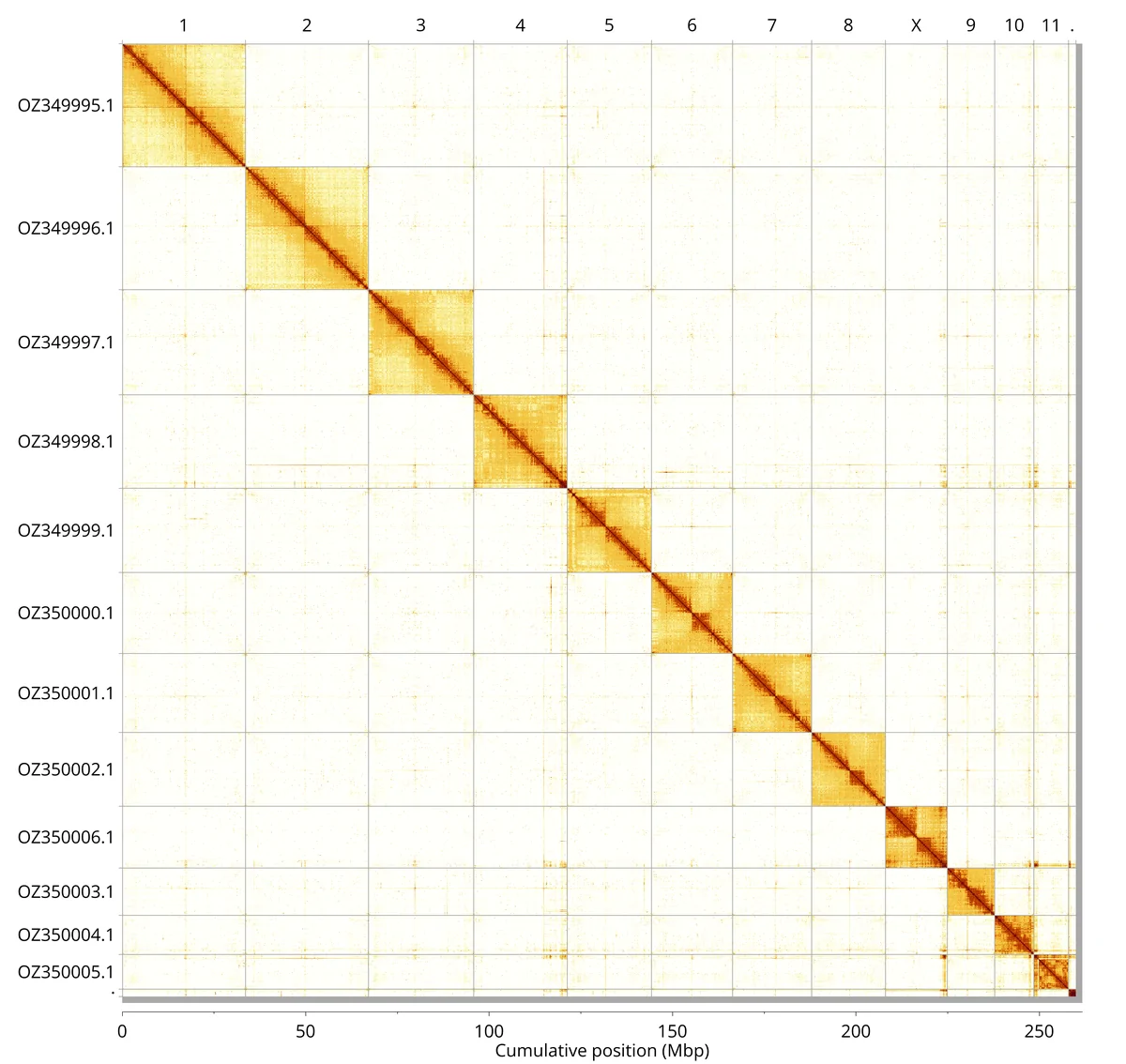
Hi-C contact map of the *S. bipustulatum* primary assembly. Assembled chromosomes are shown in order of size and labelled along the axes, with a megabase scale shown below. The plot was generated using PretextSnapshot.

**Table 3.** Chromosomal pseudomolecules in the primary genome assembly of *S. bipustulatum* icSphBipu4

**INSDC accession**	**Molecule**	**Length (Mb)**	**GC%**
OZ349995.1	1	33.58	31
OZ349996.1	2	33.43	31
OZ349997.1	3	28.62	31
OZ349998.1	4	25.47	31.50
OZ349999.1	5	22.90	31
OZ350000.1	6	22.09	31.50
OZ350001.1	7	21.47	31
OZ350002.1	8	20.12	31.50
OZ350003.1	9	12.91	32.50
OZ350004.1	10	10.62	34
OZ350005.1	11	9.46	33.50
OZ350006.1	X	16.83	32.50
OZ350007.1	Y	2.11	35.50

Chromosome painting shows the distribution of Coleoptera ancestral linkage groups assignments across the chromosome-level scaffolds (Figure 4).

**Figure 4.**
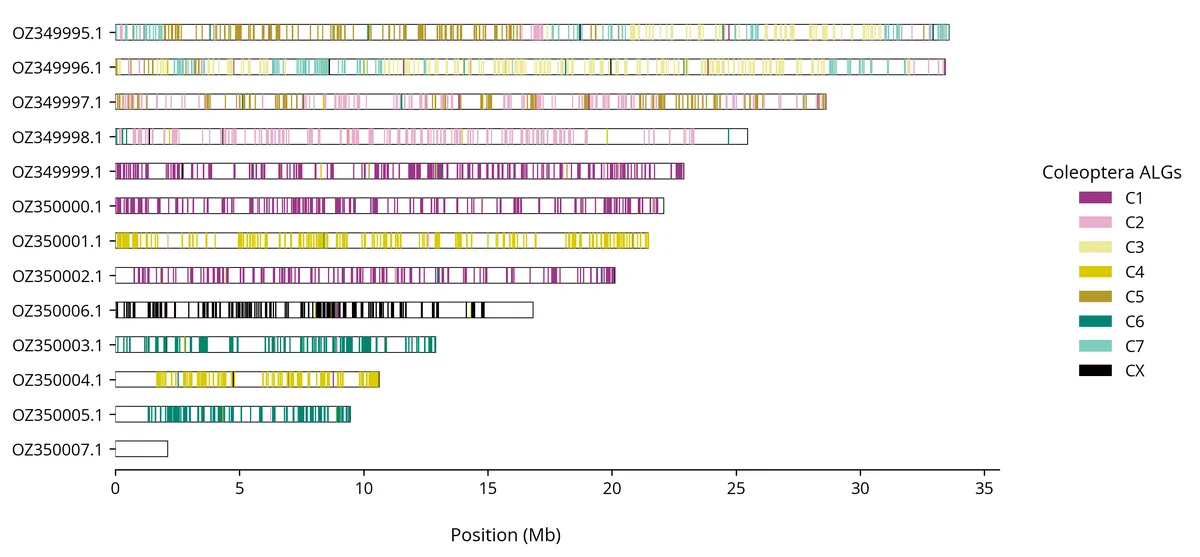
Coleoptera ancestral linkage groups painting of the genome assembly of *S. bipustulatum*. Chromosomes are drawn to scale, with coloured bars marking the positions of complete coleoptera_odb12 BUSCO genes. Each BUSCO gene is coloured by its assignment to one of the 8 Coleoptera ALGs (C1–C7 and CX).

### Assembly quality metrics

The combined primary and alternate assemblies achieve an estimated QV of 55.9. The *k*-mer completeness is 76.43% for the primary assembly, 70.33% for the alternate haplotype, and 99.57% for the combined assemblies (Figure 5).

**Figure 5.**
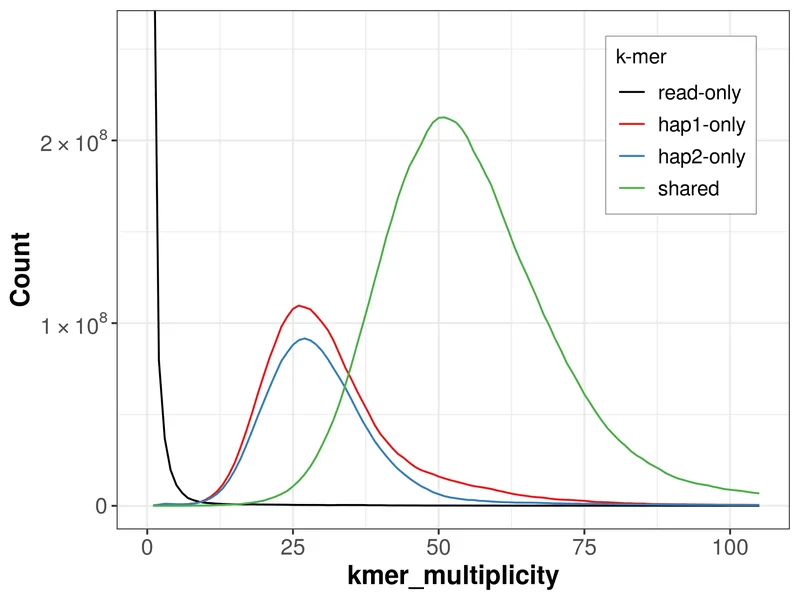
Evaluation of *k*-mer completeness using MerquryFK. This plot illustrates the recovery of *k*‐mers from the original read data in the primary assembly, the alternate haplotype assembly and the combined assemblies. The horizontal axis represents *k*‐mer multiplicity, and the vertical axis shows the number of *k*‐mers. The black curve represents *k*‐mers that appear in the reads but are not assembled. The green curve corresponds to *k*‐mers shared by both haplotypes, and the red and blue curves show *k*‐mers found only in one of the haplotypes.

BUSCO v.6.0.0 analysis using the endopterygota_odb10 reference set ( *n* = 2,124) identified 98.9% of the expected gene set (single = 97.6%, duplicated = 1.2%).

The snail plot in Figure 6 provides a summary of assembly contiguity, cumulative scaffold count, sequence composition and BUSCO completeness for the primary assembly ( Challis & Blaxter, 2026). The relative sizes of the longest scaffold, scaffold N50 and N90, together with the dark-grey scaffold-length distribution, show how much of the assembly is contained in long scaffolds. The outer composition track shows variation in GC, AT and N content, while the central track shows the cumulative scaffold count and the BUSCO inset summarises gene-set completeness.

The blob plot in Figure 7 displays scaffolds from the primary assembly by GC proportion and base coverage. Marker area is proportional to scaffold length, and colour indicates taxonomic assignment ( Challis *et al.*, 2020).

**Figure 6.**
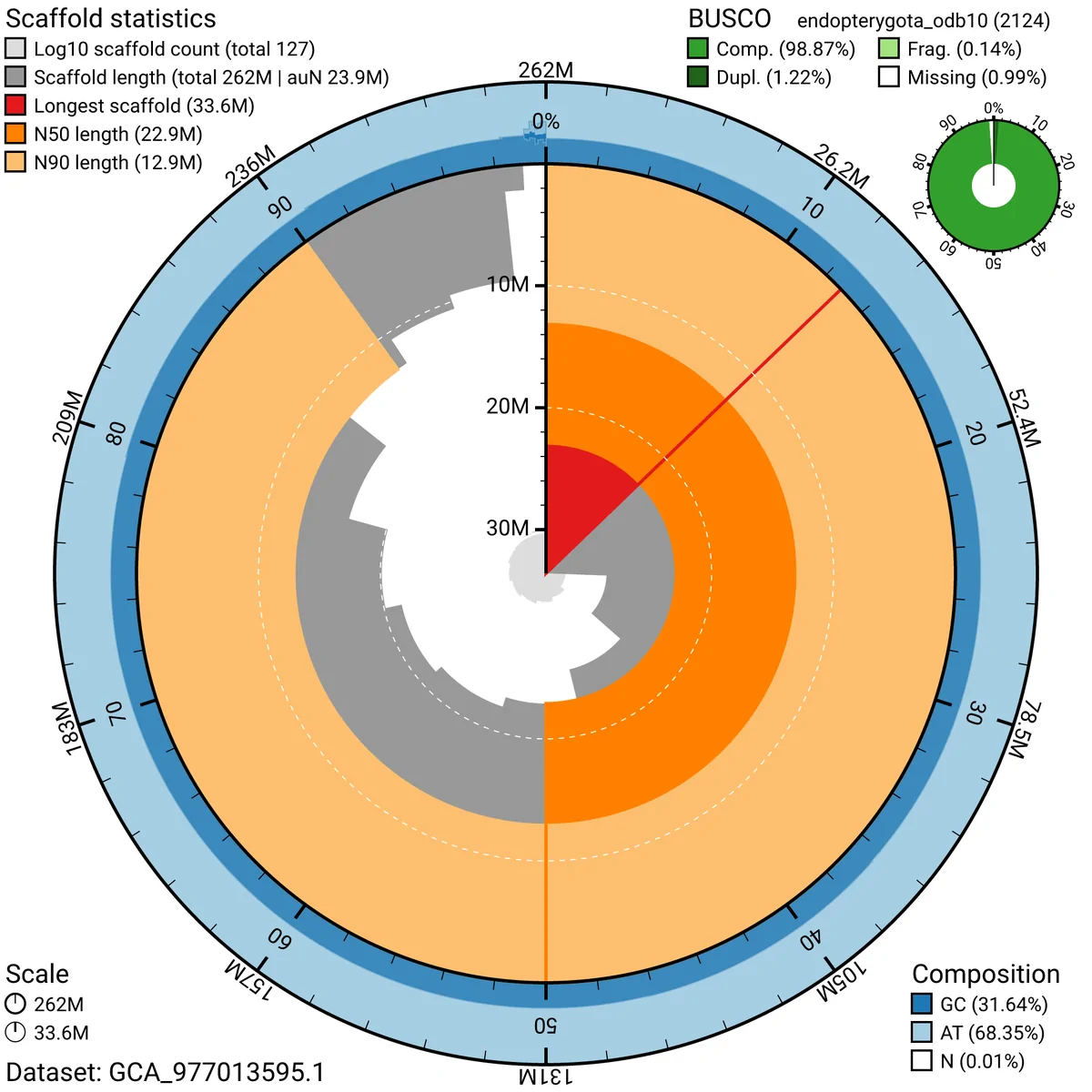
Assembly metrics for icSphBipu4.1, the primary assembly. The BlobToolKit snail plot provides an overview of assembly metrics and BUSCO gene completeness. The circumference represents the length of the primary assembly, and the main plot is divided into 1,000 bins around the circumference. The outermost blue tracks display the distribution of GC, AT, and N percentages across the bins. Scaffolds are arranged clockwise from longest to shortest and are depicted in dark grey. Scaffold length is shown on a linear radial scale, scaled to the longest scaffold. Red shading indicates the longest scaffold, and the deeper orange and pale orange shading represent the N50 and N90 lengths. The summary statistics include scaffold auN, the area under the scaffold Nx curve. The light grey spiral at the centre shows the cumulative scaffold count on a logarithmic scale. A summary of complete, fragmented, duplicated, and missing BUSCO genes in the endopterygota_odb10 set is presented at the top right. The assembly can be explored interactively in the BlobToolKit viewer.

**Figure 7.**
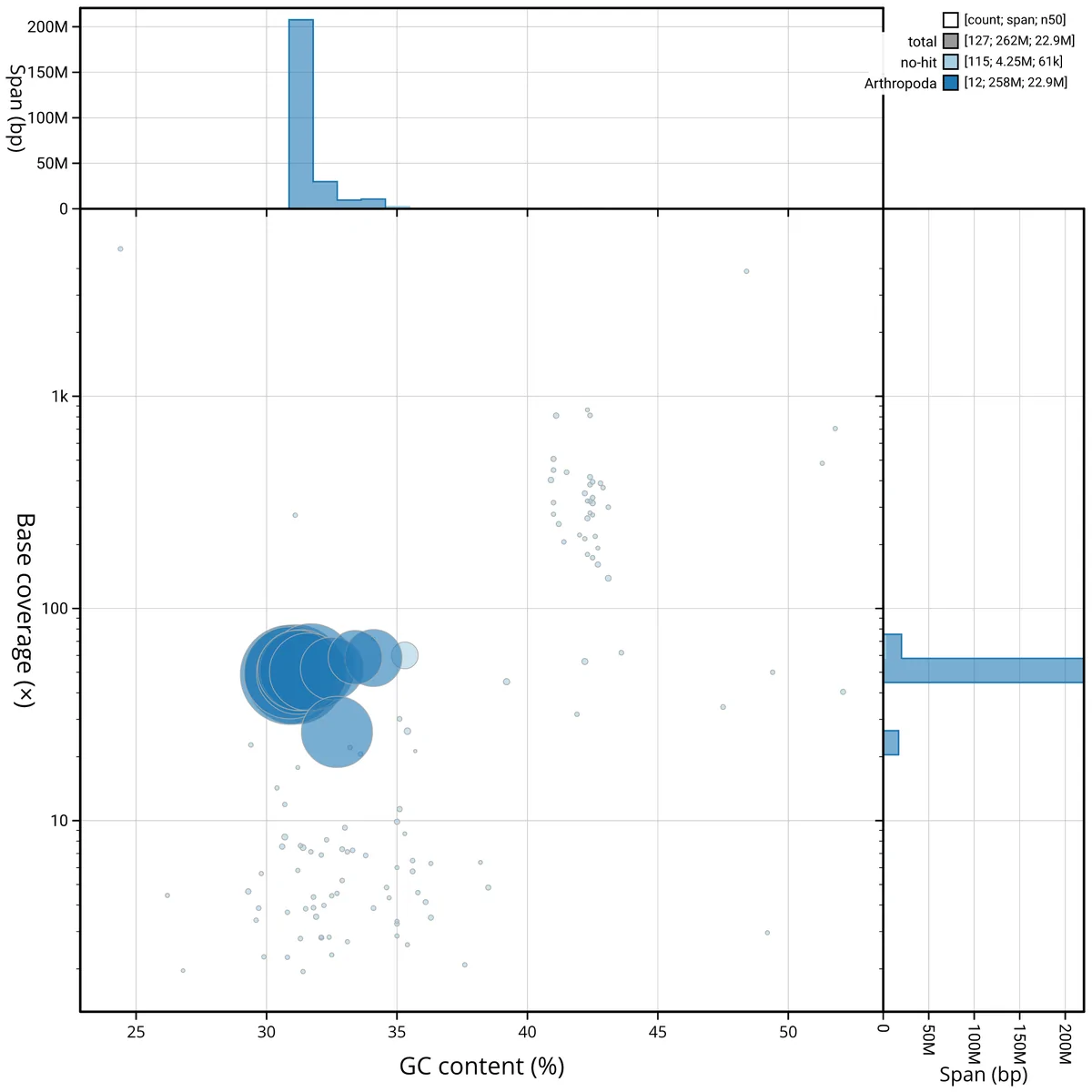
BlobToolKit blob plot for icSphBipu4.1. The plot shows base coverage (vertical axis) and GC content (horizontal axis). Circles represent scaffolds; marker area is proportional to scaffold length, and colour indicates taxonomic assignment. The histograms along the axes display the total length of sequences distributed across different levels of coverage and GC content. The assembly can be explored interactively in the BlobToolKit viewer.

Table 4 lists the assembly metric benchmarks adapted from Rhie *et al.*. (2021) and the Earth BioGenome Project (EBP) Report on Assembly Standards. The three-part EBP metric, calculated for the primary assembly, is **6.C.Q56**.

**Table 4.** Earth BioGenome Project summary metrics for the *S. bipustulatum* assembly

**Measure**	**Value**	***Benchmark***
EBP summary (primary)	6.C.Q56	*6.C.Q40*
Contig N50 length	4.12 Mb	*> 1 Mb*
Scaffold N50 length	22.90 Mb	*= chromosome N50*
Consensus quality (QV)	Primary: 56.9; alternate: 55.1; combined: 55.9	*≥ 40*
*k*-mer completeness	Primary: 76.43%; alternate: 70.33%; combined: 99.57%	*> 90%*
BUSCO	C:98.9% [S:97.6%, D:1.2%], F:0.1%, M:1.0%, n:2,124	*S > 90%; D < 5%*
Percentage of assembly assigned to chromosomes	99.19%	*≥ 90%*

**Notes:** The EBP summary uses log10(Contig N50); chromosome-level (C) or log10(Scaffold N50); Q (Merqury QV). BUSCO v6.0.0 using the endopterygota_odb10 lineage dataset: C=complete; S=single-copy; D=duplicated; F=fragmented; M=missing; n=orthologues.

### Data availability

European Nucleotide Archive: Sphaeridium bipustulatum, icSphBipu. Accession number PRJEB92312; https://identifiers.org/ena.embl/PRJEB92312. The genome sequence is released openly for reuse. The *S. bipustulatum* genome sequencing initiative is part of the Darwin Tree of Life Project ( PRJEB40665) and the Sanger Institute Tree of Life Programme ( PRJEB43745). All raw sequence data and the primary and alternate assemblies have been deposited in INSDC databases. The genome will be annotated using available RNA-Seq data and presented through the Ensembl pipeline at the European Bioinformatics Institute. Raw data and assembly accession identifiers are reported in Table 1 and Table 2.

Production code used in genome assembly at the WSI Tree of Life is available at https://github.com/sanger-tol. Table 5 lists software versions used in this study.

**Table 5.** Software versions and sources used for *S. bipustulatum*

**Software**	**Version**	**Source**
BLAST	2.14.0	https://ftp.ncbi.nlm.nih.gov/blast/executables/blast+/
BlobToolKit	4.4.6	https://github.com/genomehubs/blobtoolkit
BlobTk (hosted BlobDir)	0.5.1	https://github.com/genomehubs/blobtk
BlobTk (snail and blob plot renderer)	0.8.3	https://github.com/genomehubs/blobtk
BUSCO	6.0.0	https://gitlab.com/ezlab/busco
busco-alg-painter	0.1.0	https://github.com/Karenvn/busco-alg-painter
bwa-mem2	2.2.1	https://github.com/bwa-mem2/bwa-mem2
DIAMOND	2.1.8	https://github.com/bbuchfink/diamond
fasta_windows	0.2.4	https://github.com/tolkit/fasta_windows
FastK	1.2	https://github.com/thegenemyers/FASTK
GenomeScope	2.1.0	https://github.com/tbenavi1/genomescope2.0
Gfastats	1.3.6	https://github.com/vgl-hub/gfastats
Hifiasm	0.19.8-r603	https://github.com/chhylp123/hifiasm
HiGlass	1.13.4	https://github.com/higlass/higlass
MerquryFK	1.1.0-c1	https://github.com/thegenemyers/MERQURY.FK
Minimap2	2.28-r1209	https://github.com/lh3/minimap2
MitoHiFi	3.2.2	https://github.com/marcelauliano/MitoHiFi
MultiQC	1.14; 1.17 and 1.18	https://github.com/MultiQC/MultiQC
Nextflow	24.10.4	https://github.com/nextflow-io/nextflow
PretextSnapshot	0.0.6	https://github.com/sanger-tol/PretextSnapshot
PretextView	1.0.3	https://github.com/sanger-tol/PretextView
samtools	1.21	https://github.com/samtools/samtools
sanger-tol/ascc	0.1.0	https://github.com/sanger-tol/ascc
sanger-tol/blobtoolkit	v0.9.0	https://github.com/sanger-tol/blobtoolkit
sanger-tol/curationpretext	1.4.2	https://github.com/sanger-tol/curationpretext
Seqtk	1.4-r122	https://github.com/lh3/seqtk
Singularity	3.9.0	https://github.com/sylabs/singularity
TreeVal	v1.4.0	https://github.com/sanger-tol/treeval
YaHS	1.2.2	https://github.com/c-zhou/yahs

### Author information

Contributors are listed at the following links:

- Members of the University of Oxford and Wytham Woods Genome Acquisition Lab
- Members of the Darwin Tree of Life Barcoding collective
- Members of the Wellcome Sanger Institute Tree of Life Management, Samples and Laboratory team
- Members of Wellcome Sanger Institute Scientific Operations – Sequencing Operations
- Members of the Wellcome Sanger Institute Tree of Life Core Informatics team
- Members of the Tree of Life Core Informatics collective
- Members of the Darwin Tree of Life Consortium

### Wellcome Sanger Institute – Legal and Governance

The materials that have contributed to this genome note have been supplied by a Darwin Tree of Life Partner. The submission of materials by a Darwin Tree of Life Partner is subject to the **‘Darwin Tree of Life Project Sampling Code of Practice’**, which can be found in full on the Darwin Tree of Life website. By agreeing with and signing up to the Sampling Code of Practice, the Darwin Tree of Life Partner agrees they will meet the legal and ethical requirements and standards set out within this document in respect of all samples acquired for, and supplied to, the Darwin Tree of Life Project. Further, the Wellcome Sanger Institute employs a process whereby due diligence is carried out proportionate to the nature of the materials themselves, and the circumstances under which they have been/are to be collected and provided for use. The purpose of this is to address and mitigate any potential legal and/or ethical implications of receipt and use of the materials as part of the research project, and to ensure that in doing so we align with best practice wherever possible. The overarching areas of consideration are:

- Ethical review of provenance and sourcing of the material
- Legality of collection, transfer and use (national and international)

Each transfer of samples is further undertaken according to a Research Collaboration Agreement or Material Transfer Agreement entered into by the Darwin Tree of Life Partner, Genome Research Limited (operating as the Wellcome Sanger Institute), and in some circumstances, other Darwin Tree of Life collaborators.
