# Supplementary material for: The genome sequence of the Water scavenger beetle, Sphaeridium bipustulatum Fabricius, 1781 (Coleoptera: Hydrophilidae)
Source: Wellcome Open Res. 2026 Sep 7;11:608. [Version 1] doi: 10.12688/wellcomeopenres.27731.1 (PMC13669573; doi:10.12688/wellcomeopenres.27731.1)
Supplement: Peer review history

# Reviewer report: Reviewer response for version 1

**DOI:** 10.21956/wellcomeopenres.30522.r170416

**Published:** 2026-10-15

**Authors:** Ikechukwu Eugene Onah

**Affiliations:** 1 University of Nigeria, Nsukka, Enugu, Nigeria

The article “The genome sequence of the Water scavenger beetle, *Sphaeridium bipustulatum *Fabricius, 1781 (Coleoptera: Hydrophilidae)” is well written in clear and understandable language. The background presented the morphological identity, geographic distribution and habitats and habits of *Sphaeridium bipustulatum.  *The methodology is clear and straightforward which can be easily reproduced. However, where it was stated that the sample was dissected from the specimen and stored in ethanol, the authors should state the percentage of the ethanol used or whether it was absolute ethanol.

 Also, to ensure consistency, I suggest that where it was stated in the methodology that DNA was assessed with a NanoDrop spectrophotometer and a Qubit Fluorometer that the Manufacturer and country should be stated. This was done for some other machines in the manuscript e,g. Revio instrument (Pacific Biosciences, California, USA).

 The library preparation, genome assembly followed standard procedures and the results reflect perfectly what was done.

 Under Assembly statistics after Table two, there is a place the authors wrote HiC map. However, throughout the work there has always been a hyphen after Hi, that is Hi-C map. Let there be consistency in the format of writing the terminology.

 The article “The genome sequence of the Water scavenger beetle, *Sphaeridium bipustulatum *Fabricius, 1781 (Coleoptera: Hydrophilidae)” is well written in clear and understandable language. The background presented the morphological identity, geographic distribution and habitats and habits of *Sphaeridium bipustulatum.  *The methodology is clear and straightforward which can be easily reproduced. However, where it was stated that the sample was dissected from the specimen and stored in ethanol, the authors should state the percentage of the ethanol used or whether it was absolute ethanol.

 Also, to ensure consistency, I suggest that where it was stated in the methodology that DNA was assessed with a NanoDrop spectrophotometer and a Qubit Fluorometer that the Manufacturer and country should be stated. This was done for some other machines in the manuscript e,g. Revio instrument (Pacific Biosciences, California, USA).

 The library preparation, genome assembly followed standard procedures and the results reflect perfectly what was done.

 Under Assembly statistics after Table two, there is a place the authors wrote HiC map. However, throughout the work there has always been a hyphen after Hi, that is Hi-C map. Let there be consistency in the format of writing the terminology.

Are the datasets clearly presented in a usable and accessible format, and the assembly and annotation available in an appropriate subject-specific repository?

Yes

Are sufficient details of the sequencing and extraction, software used, and materials provided to allow replication by others?

Yes

Are the rationale for sequencing the genome and the species significance clearly described?

Yes

Are the protocols appropriate and is the work technically sound?

Yes

Reviewer Expertise:

My area of research is entomology. I use DNA barcoding and genomics to identify, document and assess insect biodiversity.

I confirm that I have read this submission and believe that I have an appropriate level of expertise to confirm that it is of an acceptable scientific standard.

# Reviewer report: Reviewer response for version 1

**Published:** 2026-09-07

**Authors:** Automated Benchmarking

**Affiliations:** 1 Wellcome Sanger Institute, Hinxton, UK

As this article is a Genome Note, Automated Benchmarking has been used to determine whether the reported assembly passes three defined checks established by the Earth BioGenome Project: the raw and assembled data are publicly available, the Earth BioGenome Project three-part metric is met, and the BUSCO score passes the stated threshold. For Sphaeridium bipustulatum (icSphBipu4), all three checks passed. Data available: pass. ENA study PRJEB92312, raw-read accessions ERR15282593, ERR15286579, and ERR17468150, and assembly accessions GCA_977013595.1 and GCA_977013195.1 are public. Earth BioGenome Project three-part metric: pass. The primary assembly is reported as 6.C.Q56, meeting the 6.C.Q40 reference standard. BUSCO: pass. The primary assembly, which is proposed as the reference assembly for this species, has 98.9% complete BUSCOs (97.6% single-copy and 1.2% duplicated). It meets the target of greater than 90% complete and single-copy BUSCOs. This decision uses values reported in the Genome Note and public accession records.

Reviewer Expertise:

NA

I confirm that I have read this submission and believe that I have an appropriate level of expertise to confirm that it is of an acceptable scientific standard.
